# Monitoring and Evaluating Progress towards Universal Health Coverage in Tanzania

**DOI:** 10.1371/journal.pmed.1001698

**Published:** 2014-09-22

**Authors:** Gemini Mtei, Suzan Makawia, Honorati Masanja

**Affiliations:** 1 Ifakara Health Institute, Dar es Salaam, Tanzania

## Abstract

This paper is a country case study for the Universal Health Coverage Collection, organized by WHO. Gemini Mtei and colleagues illustrate progress towards UHC and its monitoring and evaluation in Tanzania.

*Please see later in the article for the Editors' Summary*

This paper is part of the PLOS Universal Health Coverage Collection. This is the summary of the Tanzania country case study. The full paper is available as Supporting Information file Text S1.

## Background

Achieving universal health coverage (UHC)—defined as access to needed health services to all and protection against financial risks arising from paying for health services [1]—is among the top priorities of reform agendas across many countries. Provision of health services should be determined by individuals' need rather than their ability to pay and, at the same time, utilization of services by those seeking health care should not impose the risk of financial catastrophe [2],[3]. In addition, paying for health care should be administered in an equitable manner whereby individuals with a higher ability to pay contribute a relatively higher share of their income to health financing compared to those individuals with a lower ability to pay [3]. Tanzania has been making efforts towards UHC starting with the abolition of user fees soon after independence in 1967, before their reintroduction in the early 1990s, and the introduction of health insurance schemes in early 2000.

## Universal Health Coverage: The Policy Context

Immediately after independence in 1961, Tanzania adopted a free health care policy that lasted until the early 1990s when user fees were reintroduced, accompanied by exemptions and waivers for the poor [4]. In 1999 the National Health Insurance Fund (NHIF) for formal public sector employees was introduced [5], followed by the community health fund (CHF) enacted in 2001 to cover the informal sector of the population (such as farmers, unregistered business owners, and the unemployed) [6]. In 2007 the country adopted a ten year (2007–2017) primary health care development program (PHCDP), whose main objective is improvement of the primary health care delivery system by increasing the number of health facilities, especially in rural areas [7]. Currently the government is in the process of developing its first National Health Financing Strategy (HFS), which stipulates the intention of developing a health financing system that will guarantee access to needed care for all and provide financial protection against payments for health care.

## Monitoring and Evaluation for Universal Health Coverage

**Figure 1:**
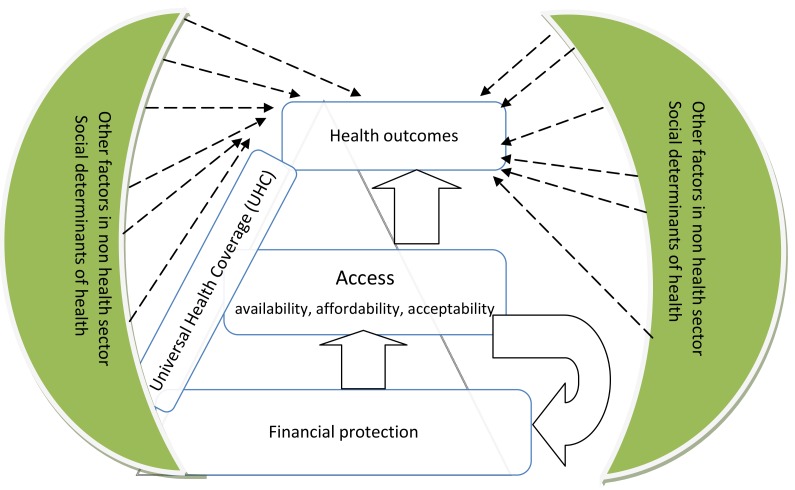
Universal health coverage assessment framework.

The assessment of progress in financial protection used data from the National Household Budget Survey 2007, the Strategies for Health Insurance for Equities in Less Developed Countries (SHIELD) survey 2008, and the National Panel Survey (NPS) 2010/2011. Access indicators were derived from the analytical report prepared for medium term review of the health sector strategic plan 2009–2015. Indicators were selected on the basis of availability of data, their priorities in the health sector strategic plans, and the degree to which the indicator addresses a critical health system problem in Tanzania (e.g., availability of human resources for health). The study uses the framework presented in Figure 1 to assess progress towards UHC. The analysis starts by evaluating the extent to which needed services are accessible to the wider population; and then assesses the degree to which financial protection is guaranteed. The extent to which access and financial protection translate into improvement in health outcomes (e.g., morbidity and mortality) is also explored.

## Progress towards Universal Health Coverage in Tanzania

Out-of-pocket (OOP) payments account for about 2% of people's income. About 2% of the population incurs catastrophic health care expenditures and 1% becomes impoverished because of OOP payments. Although the observed levels of incidences of catastrophic events and impoverishment are still low in Tanzania compared to other low- and middle-income countries [8],[9], they are still unacceptable because the main purpose of UHC is to protect every individual seeking health care against the hardships and impoverishing consequences of OOP payments.

The burden of OOP payments is significantly large among the poorest segment of the population as indicated by the negative Kakwani index (see Text S1, Table S3). The Kakwani index is a measure of how health care payments deviate from proportionality. The index is negative if the financing source is regressive, positive if it is progressive, and equal to zero if it is proportional. Only 15% of the population is covered by health insurance schemes. About 40% of total health sector financing is from external sources while OOP payments account for about 30% of total health sector resources. The contribution of prepayment health insurance schemes in total financing is insignificant.

Nearly every child has access to immunization services in Tanzania (Text S1, Table S4). Challenges remain in improving access to maternal services, especially in antenatal care visits and skilled birth deliveries, as these levels are still very low. Work is also needed in ensuring that health facilities have sufficient human resources and essential drugs and medical supplies.

The country has achieved significant improvements in infant and under-five mortality. Under-five mortality decreased from 112 deaths per 1,000 live births in 2005 to 81 deaths in 2010. Infant mortality decreased from 68 deaths to 51 deaths during the same period. A challenge remains in reducing maternal mortality following a small decrease from 578 deaths in 2005 to 468 deaths in 2010. HIV prevalence is about 6% and, with the current interventions to control the spread of this disease, there are high prospects of achieving the United Nations Millennium Development Goal (MDG) targets by 2015. Although the proportion of households owning insecticide treated nets is large, malaria prevalence among outpatients is still high at 34% (see Text S1, Table S5).

## Conclusions and Recommendations

To achieve the goal of UHC, it is important for Tanzania to expand health insurance coverage through mandatory contributions to health insurance pools. Expansion of health insurance coverage will enhance financial protection among those who use services and also increase access to needed services, thereby translating into improved health status.

## Supporting Information

Text S1 The full country case study for Tanzania.
(DOCX)

## Abbreviations

OOP: out-of-pocket
UHC: universal health coverage
