# Supplementary material for: Monitoring and Evaluating Progress towards Universal Health Coverage in Tanzania
Source: PLoS Med. 2014 Sep 22;11(9):e1001698. doi: 10.1371/journal.pmed.1001698 (PMC4171093; doi:10.1371/journal.pmed.1001698)
Supplement: Text S1 — The full country case study for Tanzania. (DOCX) [file pmed.1001698.s001.docx]

**Full Case Study: Monitoring and evaluating progress towards Universal Health Coverage in Tanzania**

Gemini Mtei^1^*, Suzan Makawia^1^ and Honorati Masanja^1^

^1^ Ifakara Health Institute, Ifakara, Tanzania

*Corresponding author: Gemini Mtei

Email: gmtei@ihi.or.tz

**This paper is the full country case study to accompany the summary paper “Monitoring and evaluating progress towards Universal Health Coverage in Tanzania” that is part of the Universal Health Coverage Collection. Not commissioned; externally reviewed.**

**Abstract:**

**Background**

Achieving universal health coverage (UHC) is currently the main global agenda for health systems reforms. Countries have been making efforts in expanding health insurance coverage and improve health service availability to achieve UHC. Tracking progress over time is important for countries to plan future improvements. Most countries evaluate progress towards UHC through assessing performance in access and financial protection indicators but limited efforts have been made to integrate the performance in health outcome when assessing progress towards UHC. This article adopts a comprehensive three dimensional approach to assess progress towards UHC in Tanzania.

**Methods and Findings**

The analysis uses access, financial protection and health outcome indicators extracted from a number of secondary data sources including Household Budget Survey, National Panel Survey, Demographic and Health Survey, Health and Demographic Surveillance Systems, and Health Management Information System. Findings show that about 2% of the population suffers catastrophic health expenditure and another 1% is pushed below poverty line due to direct health care payments. The country is doing well in a number of child access indicators but still facing challenges in the improvement of maternal health and other health systems indicators such as availability of human resources for health, health facilities, drugs and medical supplies. There has been an improvement in under-five mortality but maternal mortality and control of malaria morbidity need more attention.

**Conclusions**

It is important for Tanzania to expand health insurance coverage through mandatory contributions to health insurance pools if the goal of UHC is to be realized. Expansion of health insurance coverage will help to improve financial protection among those who use services and also improve access to needed services hence translating to improved health status. Future assessments of the progress towards UHC need to incorporate an evaluation of the progress in health outcome indicators in addition to access and financial protection indicators. This broad approach will help to give a more comprehensive picture of the progress towards UHC.

**Summary Points:**

1. It is important to assess performance in financial protection, access and health outcomes when monitoring progress towards UHC
2. Progress in financial protection is better assessed by analyzing the incidence of catastrophic spending and impoverishment caused by direct out-of-pocket payments for health care
3. It is important to increase the share of domestic health funding and reduce dependence on out-of-pocket payments and donors if the UHC goal is to be achieved
4. Frequency assessment of progress towards UHC is important but require investment in health information system to be able to generate good data to undertake this exercise.

**1. Background**

Achieving universal health coverage (UHC), defined as access to needed health services to all and protection against financial risks arising from incurring medical expenses [1], is among top priorities of reform agenda across countries. Provision of health services need to be determined by individuals’ need rather than ability to pay and at the same time utilization of services by those with health care needs should not impose the risk of financial catastrophe [2,3]. In addition, payments to health care need to be arranged in an equitable manner whereby those with high ability to pay pays a relative higher share of their income to health financing compared to those with low ability to pay [3]. Countries need to frequently assess their progress towards achieving UHC by evaluating how the health system performs in relation to core indicators of access and financial protection. In its broad terms access is comprised of three main aspects, namely availability, affordability and acceptability [4]. In order to have effective access health care facilities have to be close to people and should have all necessary medical supplies to be able to address the needs for health care. Further, services have to be affordable, meaning that individuals should not face financial barriers when using health care. The affordability aspect of access has a close link to financial protection, another arm of UHC. In addition health care services should be provided in a manner that will be acceptable to the community. In this case, the design of health care provision system should take into consideration the cultures and norms of the surrounding communities. There are other two additional dimensions, accessibility and adequacy that have previously been included when assessing access [5] but to a large extent the two are captured within the three dimensions explained above. On financial protection, individuals are not expected to be deterred from the consumption of other basic services as a result of paying for health care services. In this case, health services are considered to be a right for each individual with need with imposing financial risk to the person who seeks care. A significant proportion of the population worldwide is denied access to other basic necessities of life as a result of paying for health care services and a number of people are pushed into poverty due to medical expenditures [6,7]. It is widely acknowledged that achieving the goal of universal coverage is a progress rather than a static situation [8] and it is currently hard to find a country that can be claimed to have achieved universal coverage. Almost every country face availability, utilization, quality and financial protection challenges that need progressive improvement. Practically, countries need to set a number of UHC minimal achievable targets, based on resources available and country priorities, and monitor and evaluate the progress towards achieving those targets, and keep on improving and add higher level targets as situation allows.

Indicators used to monitor and evaluate progress towards UHC normally focus on health care financing and access to health care. The two dimensions of financing and access is what is actually been given attention in the definition of UHC. However, it is important to note that consumption of health services is not for its own sake but for the improvement of health status. For health status to be improved, access to services should be effective, meaning that consumed health services are enough and of adequate quality[4]. In this case improvement in health status of the nation is an important indicator of assessing the progress towards UHC. It is also important to note that other factors in non health sectors and socio-economic determinants have impact on health outcomes.

**2. Universal health coverage: the policy context**

Immediately after independence, Tanzania adopted a free health care policy. This was part of the Arusha declaration of 1967, which insisted on socialism and self reliance. The objective under free health care policy was to ensure that each individual in the country has free access to primary health care services. However, the ambition to provide free health care to all faced an obstacle due to economic hardship in mid 1980s [9]. These economic shocks forced the government to revert to cost sharing policy in the health sector, in 1994 [10]. Nevertheless the introduction of user fees was accompanied by exemption and waiver policy, which aimed at providing financial protection for the most vulnerable groups [10]. In 1999 the national health insurance fund (NHIF) was introduced to provide financial protection for the employees in formal public sector [11] while the community health fund (CHF) was enacted in 2001 as a prepayment scheme for the informal sector [12]. The objective of attaining UHC is also stipulated in the broad development vision 2025 [13] under the slogan health for all which aim to guarantee access to health care to all by the year 2025. The national health policy and health sector strategic plan (HSSP) III, 2008-2015 also insist on the goal of achieving UHC. The HSSP III gives strategies of achieving the Millennium Development Goals (MDGs) of reducing maternal and child mortality together with reduction in prevalence of communicable diseases such as TB, HIV/AIDS and malaria. A number of programs, such as pay for performance (P4P) and insurance card for pregnant women are currently being piloted to improve maternal and child health, which are among the key objectives of the MDGs.

In 2007 the country adopted a ten year (2007-2017) primary health care development program (PHCDP), whose main objective is improvement of primary health care delivery system by increasing the number of facilities, especially in rural areas [14]. The PHCDP intends to bring health care facilities close to people, through construction of new facilities and making sure that health care facilities provide effective care through improving availability of drugs and medical supplies together with human resources for health. Currently the government through the Ministry of Health and Social Welfare is in the process of developing National Health Care Financing Strategy (HFS) which stipulates the intention of developing health financing system that will guarantee access to needed care for all and provide financial protection against payments for health care. The HFS aims at improving health insurance coverage, especially in the informal sector, finding a better way of protecting the poor against health care payments and guaranteeing access to essential health care minimum benefit package to all.

Based on above reform experiences it is definite that Tanzania has come a long way in its efforts to achieve universal health coverage. Monitoring reforms and evaluating the extent to which they have made progress towards achieving UHC is important but at the same time is a too challenging task. One major challenge is getting good data that can be used to assess progress in financial protection and access. In Tanzania there is health management information system but in many case the data is incomplete and not of good quality to assess progress in access to health care. Further, there is no good expenditure survey that collects information on health care payments that can be used to assess financial protection. In this case effort is needed to be able to triangulate different data sources and come up with indicators that can be used to assess progress towards UHC. Selecting which indicators to use when assess progress towards UHC is another challenge. There is some agreement on which indicators to use when assessing progress in financial protection. The proportion of people incurring catastrophic payments and the proportion that falls below poverty line as a result of paying for health care are the commonly used indicators for assessing financial protection. Challenge remains on assessing progress in access to health care, and especially on deriving indicators for need of care. In practice, improving access is not only facilitated by a single intervention but a number of multiple interventions which are implemented in parallel. This imposes complexities in the evaluation and measurement of progress towards achieving UHC. Countries need to select a number of core indicators from the ongoing reforms which would help to evaluate their progress towards UHC. In Tanzania the assessment of financial protection and equity in the distribution of health care payments and health care benefit was previously done but using data from 2000/01 HBS [15,16], which is now too old. This requires an update to explore how the health system progress in terms of UHC, using a more update information. Further, previous assessments have mainly focused on financial protection and access to health care services, without paying attention on how health status is improving. Combining financing protection and access together with progress with health status would help to give a comprehensive picture of the move towards UHC.

**3. Monitoring and evaluation for UHC**

**Data**

The assessment of progress in financial protection and health system financing in general used data from the National Health Accounts 2010/11, National Household Budget Survey 2007, Strategies for Health Insurance for Equities in Less Developed Countries (SHIELD) survey 2008 and National Panel Survey (NPS) 2010/11. Indicators on access to services were assessed using a number of secondary data sources as shown in Table S1. Health outcome indicators were mainly extracted from the analytical report prepared for medium term review of the health sector strategic plan 2009-2015. The medium term review report utilized information from the Health Management Information System (HMIS), Demographic and Health Surveillance (DHS) and Health and Demographic Surveillance Systems together with information from Sentinel Panel Districts (SPD).Table S1 presents a summary description of the data sources used to get indicators that were used to assess UHC in this study. The criteria used select indicators included availability of data as the main criterion, the priority of the indicator in the health sector strategic plan 2009-2015, the degree to which the indicator addresses a critical health system problem in Tanzania (e.g. availability of human resource for health) and the extent to which the indicator is a priority in the MDGs.

| **Data source** | **Year** | **Description** | **Indicators selected** |
| --- | --- | --- | --- |
| Household Budget survey (HBS) | 2007 |  | Financing incidence of Out-of-pocket payments and general taxation, Catastrophic incidence, and Impoverishment effect |
| National Panel survey (NPS) | 2010/11 |  | Satisfaction with health services |
| Health Sector Strategic Plan 2009-2015 Medium Term Review Report^1^ | 2013 | This report collected indicators from various data sources including Demographic and Health Surveillance (DHS), Sentinel Panel of Districts (SPD), Health Management Information System (HMIS) | Access to specific services, human resources availability, and health outcome indicators |
| SHIELD survey | 2008 |  | Financing incidence of the Community Health Fund (CHF) and the National Health Insurance Fund (NHIF) |
| National Health Accounts (NHA) | 2009/10 |  | Financing contribution of different sources (government, donors, out-of-pocket payments) |

**Table S1:** Data sources used to select indicators for monitoring and measuring UHC

^1^This report collected indicators from various data sources including Demographic and Health Surveillance (DHS), Sentinel Panel of Districts (SPD), Health Management Information System (HMIS)

**Analysis methods**

This article applies a three dimensional approach in the assessment of the progress towards UHC (Figure S1). It starts by evaluating the extent to which needed services are accessible to the wider population; and then assess the degree to which financial protection is guaranteed. Accessibility in this analytical framework implies availability, affordability and acceptability of services as proposed in McIntyre, et al. [4]. Lastly the extent to which access and financial protection translates to improvement in health outcome (e.g. morbidity and mortality) is also explored. It is hypothesized that, financial protection is a key pillar under UHC while access stands between financial protection and health outcomes. Distributional issues (equity), which cut across the three dimensions, such as access to specific services across different socio-economic groups are also assessed where data is available.

Other factors in non health sector

Social determinants of health

Other factors in non health sector

Social determinants of health

Other factors in non health sector

Social determinants of health

Other factors in non health sector

Social determinants of health

**Figure S1:** UHC Assessment Framework

Although it is not common to assess progress in health outcomes when evaluating progress in UHC, the inclusion of health outcome indicators helps to assess whether; 1. Services provided are enough and 2. Services are of adequate quality. Physical availability of health facilities by itself is not enough to improve health status. Facilities need to have enough drugs and supplies and manpower that will be enough to deliver effective services. Since it is difficult to get a direct measure of effective service delivery the inclusion of health outcomes helps to indirectly assess whether services provided are adequate and of good quality. It is important to acknowledge that not only access and availability of health services but also other factors in the non health sector and social determinants have impact on health outcomes. The framework proposed in this study also takes into consideration of this fact (Figure S1) but does not go into details in analysing the impact of other social determinants on health outcomes

The assessment of the progress towards UHC is done by exploring the extent to which the country is performing in relation to a number of pre selected, access, financial protection and health outcomes indicators. Priority indicators to be evaluated in this study, especially indicators of access and health outcomes are selected based on local priorities, as specified in various policy documents, such as the Vision 2025, the National Health Policy (NHP), Primary Health Care Development Program (PHCDP), (famously known as MMAM) and Health Sector Strategic Plan (HSSP) III. As already highlighted before the criteria for selecting indicators included availability of data as the main criterion, the priority of the indicator in the health sector strategic plan 2009-2015, the degree to which the indicator addresses a critical health system problem in Tanzania (e.g. availability of human resource for health) and the extent to which the indicator is a priority in the MDGs. The available literature identifies a number of key challenges in the health sector in Tanzania that need immediate attention. These include crisis in human resources for health [17], unavailability of health services, especially drugs [17], limited health insurance coverage [16], challenges in addressing maternal mortality [18], high malaria prevalence, and limited financial protection [19]. These were used in prioritizing indicators to evaluate. This was complemented by indicators used internationally, especially the MDGs indicators, for comparability purpose. One of the challenges when assessing progress is the availability of data to explore acceptability of health care. Most of the available indicators have focused on the issues of availability and affordability of health care. We have attempted to explore accessibility in this article using satisfaction with health care as a proxy. This data is extracted from the National Panel data. There is almost international consensus on the use of proportion of people incurring catastrophic expenditure and proportion of people driven into poverty because of health care payments as the two measures of financial protection [20,21]. The two have been used in this paper to assess progress in financial protection in Tanzania. In addition, equity in health care payments and the proportion of people having health insurance coverage are used to complement the above two measures.

**4. Progress towards UHC in Tanzania**

**Distribution of health financing sources**

The Tanzania health system is financed through a number of financing sources including general taxation, out-of-pocket payments, health insurance and donors. General government expenditure on health accounts for about 40% of total financing while out-of-pocket payments also plays a significant role in health sector financing (Table S2). On average the government allocated about 11% of its total expenditure to the health in 2009/10. This is still below the Abuja target of 15% of total government budget to the health sector. Total health sector expenditure accounts for about 7% of GDP, a figure which is higher compared to the average of 6% for sub-Saharan Africa countries. Health insurance contribution to total health sector financing plays an insignificant role of about 5% of total health sector budget [19].

| **Financing indicator** | **Percent** |
| --- | --- |
| Total expenditure on health as % of GDP | 7.2 |
| Per capita government expenditure on health at PPP $ | 42 |
| General government expenditure on health (GGHE) as % of GDP | 5.4 |
| General government expenditure on health as % of total government expenditure (GGHE/GGE) | 11.1 |
| General government expenditure on health as % of total expenditure on health | 39.5 |
| Private expenditure on health as % of total health expenditure on health | 60.5 |
| External resources for health as % of total health expenditure on health | 41.2 |
| Out-of-pocket (OOP) expenditure on health as % of total expenditure on health | 31.7 |
| Out-of-pocket expenditure on health as % of GDP | 2.3 |
| Private prepaid plans on health as % of total expenditure on health | 1.5 |

**Table S2:** The structure of health financing system in Tanzania

Data Source: WHO <http://apps.who.int/nha/database/StandardReport.aspx?ID=REPORT_2_WHS>

**Progress in financial protection**

Out-of-pocket payments accounts for about 2% of people’s income. Despite this relatively low share of OOPs to income, about 2% of the population incurs catastrophic health care expenditures and 1% is impoverished due to out-of-pocket payments (Table S2). Catastrophic payments mainly affect the poorest than the rich households (Figure S2).

**Figure S2:** Distribution of catastrophic spending across socio-economic groups

Data Source: [19]

The analysis shows that the burden of OOPs is significantly large among the poorest as indicated by the negative Kakwani index in Table S3. N.B.: Kakwani index is a measure of how health care payments deviate from proportionality. The index is negative if the financing source is regressive, positive if it is progressive, and equal to zero if it is proportional.

Payments to health care through general taxation (direct and indirect taxes) are progressive, implying that it is mainly the rich who incur a higher burden of this source of health care financing.

About 15% of the total population has some form of health insurance coverage. Formal sector insurance coverage accounts for about 7.1% of the population while the informal sector insurance coverage is about 6.9%. This level of coverage in the informal sector is still very low compared to the population of more than 80% working in the informal sector. The analysis indicates that contributions to the formal sector health insurance are progressive while informal sector health insurance contributions are highly regressive (Table S3).

| **Indicator** | **Progress** | **Target/benchmark** |
| --- | --- | --- |
| Catastrophic Headcount (2007) | 1.80% | 0 |
| % of population impoverished due to OOPs (2007) | 0.80% | 0 |
| Out-of-pocket payments share of income (2007) | 2.00% | 0^1^ |
| **Kakwani indices ( 2007)** |  |  |
| Out-of-pocket | -0.07 | + |
| Direct taxes | 0.41 | + |
| Indirect tax | 0.14 | + |
| Formal sector insurance (NHIF) | 0.42 | + |
| Informal sector insurance (CHF) | -0.49 | + |
| Formal sector insurance coverage (2011) | 6.90 | 100 |
| Informal sector insurance coverage (2011) | 7.10 | 100 |

**Table S3:** Status of financial protection indicators

Data Source: [19]

^1^This might only be a true target for the poor and those who are not covered by any health insurance scheme. It might be unrealistic to have a zero target among insured because there might be cases of co-payments and exclusions that need to be paid out of pocket. However, we think in this article that individuals would be better protected if health care payments are arranged through prepayment schemes and there are zero out-of-pocket payments

**Progress in access to health care**

Nearly every child has access to immunization services in Tanzania. Challenge still remains in improving access to maternal services, especially completion of ANC visits and skilled birth deliveries, as the levels are still very low and making sure that health facilities have enough human resources and essential drugs and medical supplies (Table S1). There are still challenges in access maternal services especially completion of ANC visits and skilled birth deliveries. Only 36% of pregnant women had at least 4 ANC visits in 2012 while 58% had skilled birth attendance (Table S4).

| **Indicators (i)** | **Progress** | **Target/**  **benchmark** | **Progress relative to the target/benchmark**  **(P_i_)** |
| --- | --- | --- | --- |
| Measles immunization^a^ | 100.0% | 100.0% | 100.0% |
| DTP-Hb 3 immunization^a^ | 95.0% | 100.0% | 95.0% |
| Vit A coverage 2-doses^a^ | 60.0% | 100.0% | 60.0% |
| TT2 immunization coverage^a^ | 88.0% | 100.0% | 88.0% |
| ANC at least 4 visits^a^ | 36.0% | 100.0% | 36.0% |
| Births in health facilities^a^ | 58. 0% | 100.0% | 58.0% |
| Skilled birth attendance^a^ | 62.0% | 100.0% | 62.0% |
| Postnatal care coverage^a^ | 31.0% | 100.0% | 31.0% |
| Contraceptive prevalence rate^a^ | 27.0% | 100.0% | 27.0% |
| ITN use (children/pregnant women) ^b^ | 73.0% | 100.0% | 73.0% |
| ART coverage among those in need ^b^ | 65.0% | 100.0% | 65.0% |
| Doctors, AMO and nurses per 10,000 population ^a^ | 5.7 | 23.0 | 24. 8% |
| OP visits per capita ^c^ | 0.8 | 4.0 | 20.0% |
| Hospitals per 100,000 population ^d^ | 0.5 | 1.0 | 53.0% |
| Health centres per 50,000 population ^d^ | 0.6 | 1.0 | 65.0% |
| Dispensaries per 10,000 population ^d^ | 1.2 | 1.0 | 119.0% |
| %age of population within 6km to health facility^e^ | 75.0% | 100.0% | 75.0% |
| Proportion of population getting prescribed medicine at the facilities ^f^ | 59.0% | 100.0% | 59.0% |
| Proportion of essential drugs available in public facilities ^f^ | 76.0% | 100.0% | 76.0% |

**Table S4:** Status of selected health care access indicators

Data Sources**:**

a=Health Sector Strategic Plan (HSSP) medium term review report 2013;

b=Demographic and Health Surveillance report 2010;
c= [22];

d=HSSP;

e=Household Budget Survey 2007;

f= <http://www.twaweza.org/uploads/files/SzWAccesstoMedicinesBrief5190913EN.pdf>;

About 75% of the population live within 6km to a dispensary or health centre [23]. The assessment shows that the country is still facing challenges in increase the number of health centres and higher level facilities. In addition, the availability of human resources for health needs immediate attention in Tanzania.

There is also a challenge in making sure that health facilities are functioning properly by ensuring availability of essential drugs and supplies. The available evidence shows that about 24% of the essential drugs are not available in public health facilities and nearly 41% of the population were not able to obtain the prescribed drugs from the facilities they visited in 2013 (<http://www.twaweza.org/uploads/files/SzWAccesstoMedicinesBrief5190913EN.pdf>).

The available information also shows under utilization of outpatient health care. On average the country has less than 1 per capita outpatient visits per year compared to the proposed benchmark of 4 visits per capita per year [22]. Further, there is also evidence of poor satisfaction with health services provided in the country (Figure S3). The available information indicates that only 58% of the individuals who visited a health provider in 2010 were at least satisfied with the services provided at the visited facilities (National Panel Survey 2010).

**Figure S3:** Satisfaction with health care as a proxy for acceptability of health care

Data Source: National Panel Survey 2010

**Progress in health outcome indicators**

The country has noted a significant improvement in infant and under-five mortality rates (Table S5). Under-five mortality decreased from 112 deaths per 1000 live births in 2005 to 81 deaths in 2010. Infant mortality decreased from 68 deaths to 51 deaths during the same period. There is still a challenge in reducing maternal mortality following a small decrease from 578 deaths in 2005 to 468 deaths in 2010.

HIV prevalence is about 6% and with the current interventions to control the spread of this disease, there are high prospects of achieving the MDG targets by 2015. Although ownership of insecticide treated nets at household level and for children younger than five years of age sleeping under an ITN has increased, malaria prevalence among the OPD patients still stands at 34%.

| **Indicator** | **Progress** | **Target/benchmark** |
| --- | --- | --- |
| Life expectancy | 59.5 | NA |
| Under 5 mortality per 1000 LB | 81.0 | 64.0 |
| Infant mortality per 1000 LB | 51.0 | 38.0 |
| Child stunting rate | 35.0% | 23.3% |
| Child underweight rate | 14.0% | 14.4% |
| Maternal mortality rate per 100,000 LB | 454.0 | 133.0 |
| Fertility rate | 5.4 |  |
| HIV prevalence (2008) | 6.0% | 5.0% |
| HIV prevalence among pregnant women (2006) | 6.8% | 5.0% |
| Malaria prevalence among OPD | 34.0% | NA |

**Table S5:** Status of selected health outcome indicators

Data Source: HSSP medium term review report 2013; NA=Not Available

**5. Conclusions and recommendations**

The incidence of catastrophic health care payments and the proportion of the population falling below the poverty line as a result of direct payments when seeking health care currently stand to be better indicators in monitoring progress in financial protection as one of the objectives of UHC across countries. In Tanzania about 2% of the population incurs catastrophic health expenditure level and about 1% is pushed below poverty line due to out-of-pocket payments for health care. Although the observed levels of catastrophic and poverty incidences due to out-of-pocket payments in Tanzania are still low compared to other low and middle income countries [24,25] they are still unacceptable because the overall target of UHC is to protect every individual who needs care against catastrophic and impoverishment effects of out-of-pocket payments. It is important to note that the catastrophic and impoverishment assessments only include individuals who sought health services [26,27] and ignore the ones who due to financial barriers cannot manage to access health care. Hence the observed levels of catastrophic and impoverishment effects might under-estimate the financial protection problem. The assessment has also shown that it is mainly the poorest who suffer catastrophic effect the most; and this group is the one that bears the largest burden of out-of-pocket payments as shown by the regressive nature of this financing source. This implies that the current financial protection measures are mainly in favour of the wealth segment of the population, hence imposing a major challenge in progress towards UHC. Evidence shows that it is mainly the poorest that have the most need [16] and without guaranteeing protection for this group, the progress towards UHC will be slow. We recommend that countries need to adopt initiatives that will improve financial protection, especially through the expansion of health insurance, through increasing general tax funding, especially for the poor and expansion of risk pooling through mandatory contributions to insurance schemes. In this case those who are currently seeking care will not risk falling into poverty or denied consumption of other basic necessities and those who are in need but not able to purchase health care will be able to utilize health care services.

The assessment has shown that big progress has been observed in accessibility to targeted services for children, especially on immunization services. However, challenge remains in improving maternal services and the over whole health system improvement. The proportion of pregnant women who are attended by professional health workers is still very low and the proportion of medical staffs to population is also not encouraging. Further the number of facilities available are not enough to provide services to the population as per expected norms and at the same time the facilities are not equipped with enough drugs and supplies to meet the demand of the whole population in need. Availability of services is an important aspect if the goal of UHC is to be achieved.

Incorporating health outcome indicators in a three dimensional assessment approach helps to give a complete picture of the progress towards UHC. The assessment suggests that the improved access to children services has been effective and of good quality to be able to translated to positive health outcomes for this group. The level of under-five and infant mortality is now close to expected target which gives the hopes of achieving MDGs targets in Tanzania. In contrast, limited access to maternal related services leaves maternal mortality at a very high level, imposing doubts in achieving the MDGs targets. Limited access to contraceptive services leaves the level of fertility at a high level. Prevalence of malaria in also high in Tanzania and this might probably be a result of limited improvement in the over whole health system, in terms of availability of human resource for health, and limited availability of drugs and other medical supplies as shown in the progress of access indicators. Further, although not everyone need has got access to ART, the current initiatives have managed to maintain HIV prevalence at low level of 6%. Nevertheless this requires further improvement to be able to reach 0 HIV prevalence rate.

We argue that countries need to adopt a health system wide improvement approach instead of only limiting efforts in improving specific targeted health services such as services for under five and pregnant women and other specific disease interventions. Improving the over whole health system for example through improvement of provider network and ensure availability of drugs and medical supplies for all types of health services will have wider impact on health outcomes improvement compared to targeting only few specific services for few groups. Targeted approaches towards health system improvement might result to unintended inequities if the most at need groups will not be addressed. Improving the over whole health system gives equal opportunities of health service utilization to all individuals at need.

It is important to address the limitation that most of the available access and health outcome indicators that can be used to assess progress towards UHC are mainly related to targeted services and not for the whole health system. In this case, it is currently difficult to get good indicators for example for non-communicable diseases and other neglected illness because they are not currently standing at the top agenda of health system improvement, especially in low income countries. Government need to put more effort to make sure that resources are not only directed towards control of communicable diseases and improvement of specific disease programs but also target the improvement of the whole health system and making sure that the needs of all individuals are met. Box S1 below summarizes recommendations from this review.

| **Box S1: Recommendations**   1. It is important for countries to adopt a three dimensional approach, by assessing progress in financial protection, access and health outcomes in order to get a complete picture of the progress towards UHC 2. The incidences of catastrophic payments and the degree to which direct health care payments drive individuals into poverty are currently preferred measures of financial protection and countries need to make frequent assessment of the two in the evaluation of progress towards UHC 3. Tanzania and other low income countries need to adopt a broad systems wide reform approach instead of only focusing to specific targeted services if the goal of UHC is to be achieved. It is important to improve provider network, train and recruit more health workers and make sure that drugs and supplies are available in all facilities. 4. Increasing the level of domestic funding especially through mandatory health insurance contributions is very crucial. High dependence on out-of-pocket payments and donor funding will delay the progress towards UHC. 5. Investment in information systems is crucial in order to be able to get good data that can be used to frequently assess progress towards UHC. Currently there is limited information to assess progress in non-communicable and chronic disease indicators. It is important to capture information on such indicators in order to be able to get a complete picture of the progress towards UHC in Tanzania. |
| --- |

**References**

1. WHO (2010) World Health Report. Geneva Switzerland: WHO.

2. World Health Organization. Social health insurance: Sustainable health financing, universal coverage and social health insurance; 2005a 16-25 May; Geneva, Switzerland. WHO.

3. World Health Organization. Resolutions and decisions; 2005b 16-25 May; Geneva, Switzerland. WHO.

4. McIntyre D, Thiede M, Birch S (2009) Access as a policy relevant concept in low and middle income countries. Health Economics, Policy and Law 4: 179-193.

5. Penchansky R (1977) The concept of access: a definition. Michigan: University of Michigan.

6. Xu K, Evans DB, Carrin G, Aguilar-Rivera AM, Musgrove P, et al. (2007) Protecting households from catastrophic health spending. Health Affairs 26: 972-983.

7. Xu K, Evans DB, Kawabata K, Zeramdini R, Klavus J, et al. (2003) Household catastrophic health expenditure: a multicountry analysis. Lancet 362: 111-117.

8. Kutzin J (2013) Health financing for universal coverage and health system performance: concepts and implications for policy. Bull World Health Organ 91: 602–611.

9. Shaw RP, Ainsworth M, editors (1995) Financing health services through user fees and insurance: case studies from Sub-Saharan Africa. Washington, D.C.: The World Bank.

10. Mubyazi G (2004) The Tanzanian policy on health-care fee waivers and exemptions in practice as compared with other developing countries: evidence from recent local studies and international literature. East African journal of public health 1: 1-10.

11. United Republic of Tanzania (1999) The National Health Insurance Fund Act. The United Republic of Tanzania.

12. United Republic of Tanzania (2001) The Community Health Fund Act. Dar es Salaam: United Republic of Tanzania.

13. Planning Commission (1995) The Tanzania Development Vision 2025. Dar es salaam, Tanzania.

14. Ministry of Health and Social Welfare (2007) Primary Health Services Development Program-MMAM 2007-2017. Dar es Salaam: Ministry of Health and Social Welfare.

15. Mills A, Ally M, Goudge J, Gyapong J, Mtei G (2012) Progress towards universal coverage: the health systems of Ghana, South Africa and Tanzania. Health Policy and Planning 27: i4-i12.

16. Mtei G, Makawia S, Ally M, Kuwawenaruwa A, Meheus F, et al. (2012) Who pays and who benefits from health care? An assessment of equity in health care financing and benefit distribution in Tanzania. Health Policy and Planning 27: i23-i34.

17. Macha J, Harris B, Garshong B, Ataguba JE, Akazili J, et al. (2012) Factors influencing the burden of health care financing and the distribution of health care benefits in Ghana, Tanzania and South Africa. Health Policy and Planning 27: i46-i54.

18. National Bureau of Statistics, Opinion Research Coorporation MACRO (2010) Tanzania Demographic and Health Survey 2010. Dar es Salaam, Tanzania: NBS AND ORC Macro.

19. Mtei GJ (2012) Health care financing progressivity and household risk protection in the context of health system financing reforms in Tanzania. London: London School of Hygiene and Tropical Medicine, University of London. 392 p.

20. World Health Organization. Sustainable health financing structures and universal coverage; 2011; Geneva.

21. World Health Organization (2010) Health system financing: The path to universal coverage. Genera: World Health organization.

22. McIntyre D (2013) Measuring health service utilization and access in the context of UHC. Presentation at the 9th World Congress of the International Health Economics Association. Sydney: Global Nework for Health Equity.

23. National Bureau of Statistics (2009) Tanzania Household Budget Survey 2007. Dar es Salaam: National Bureau of Statistics, Tanzania.

24. Van Doorslaer E, O'Donnell O, Rannan-Eliya RP, Somanathan A, Adhikari SR, et al. (2007) Catastrophic payments for health care in Asia. Health Economics 16: 1159-1184.

25. van Doorslaer E, O'Donnell O, Rannan-Eliya RP, Somanathan A, Adhikari SR, et al. (2006) Effect of payments for health care on poverty estimates in 11 countries in Asia: an analysis of household survey data. Lancet 368: 1357-1364.

26. Flores G, Krishnakumar J, O'Donnell O, Van Doorslaer E (2008) Coping with health care costs: implications for the measurement of catastrophic expenditure and poverty. Health Economics 17: 1393-1412.

27. Wagstaff A, van Doorslaer E (2003) Catastrophe and impoverishment in paying for health care: with applications to Vietnam 1993-1998. Health Economics 12: 921-934.
